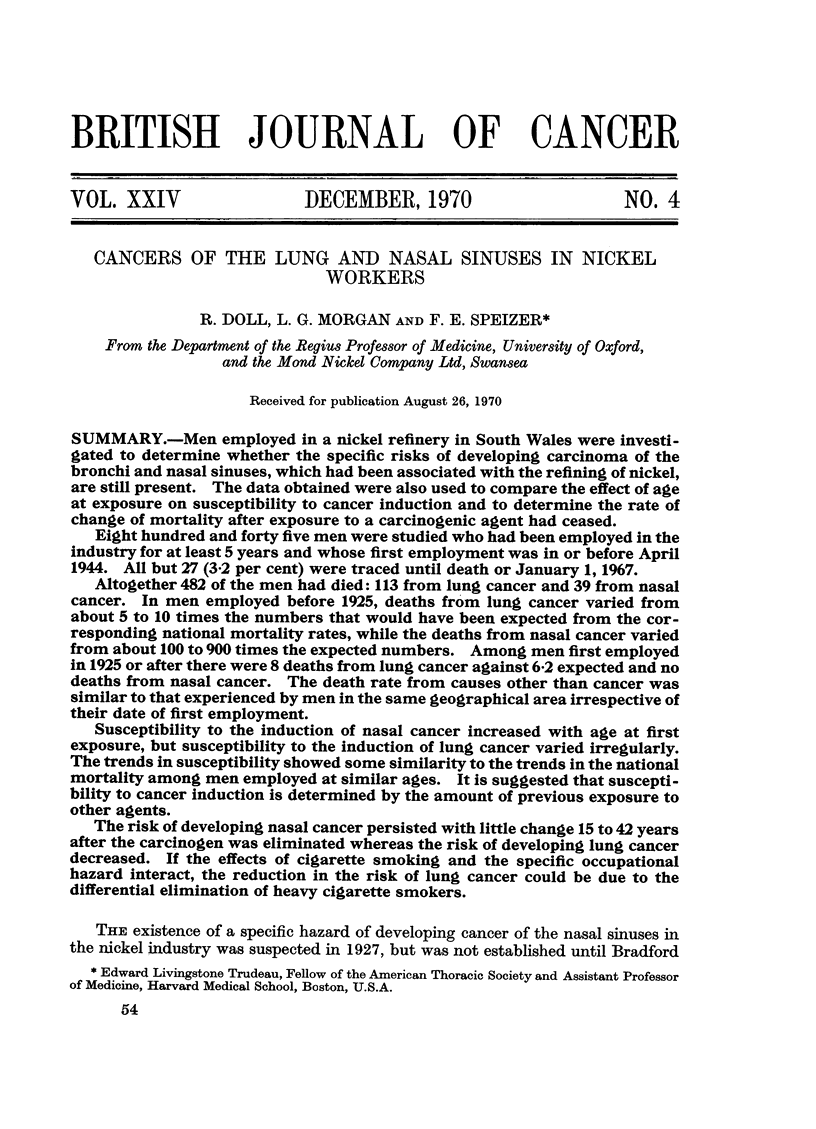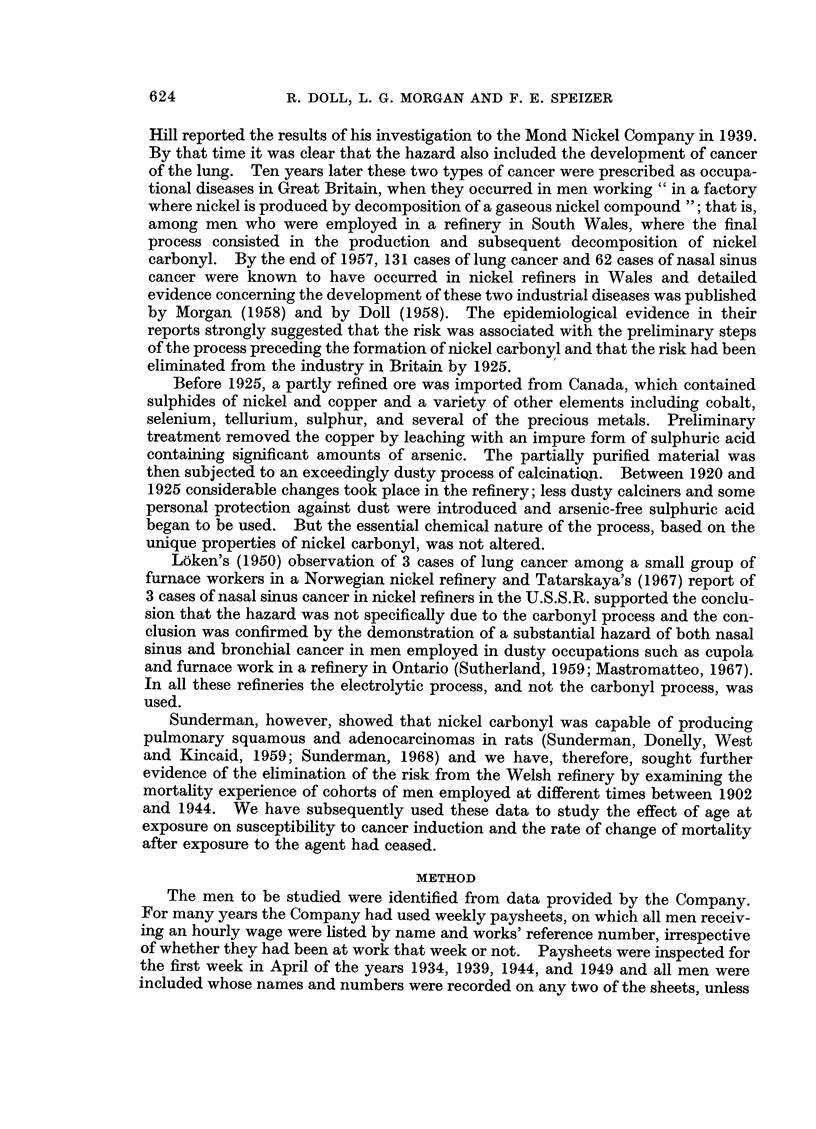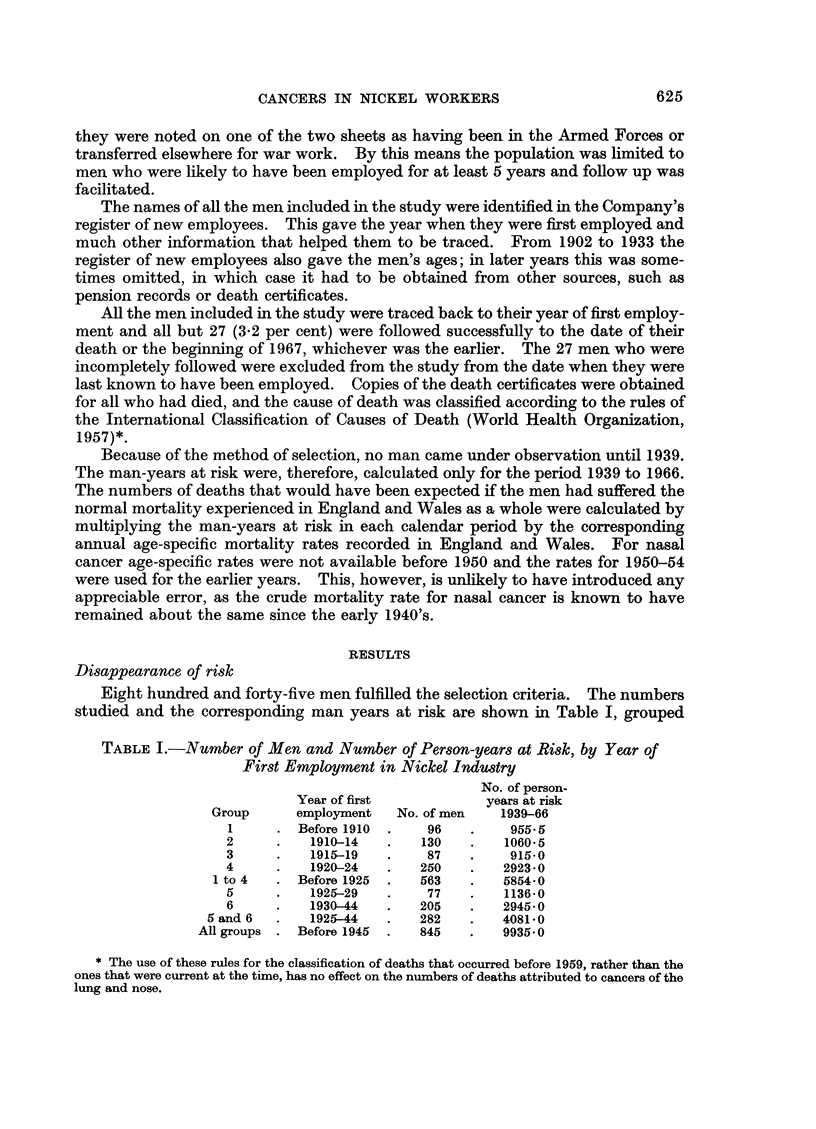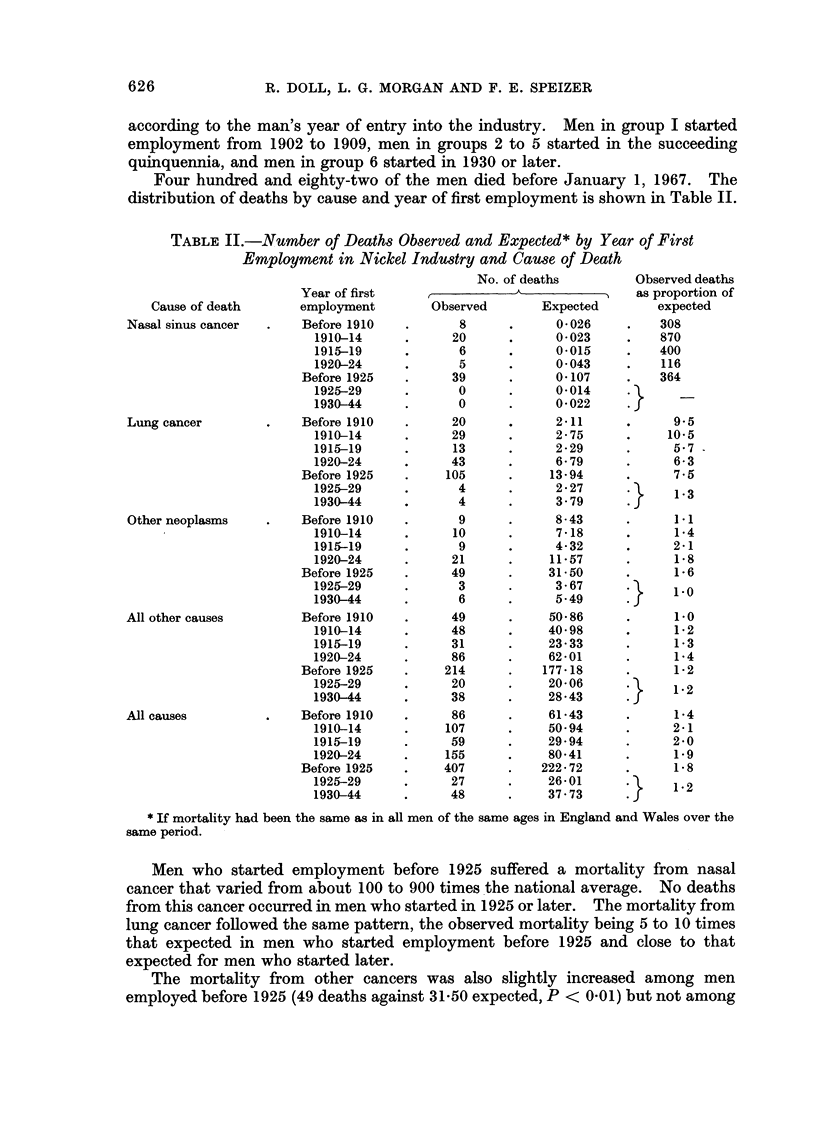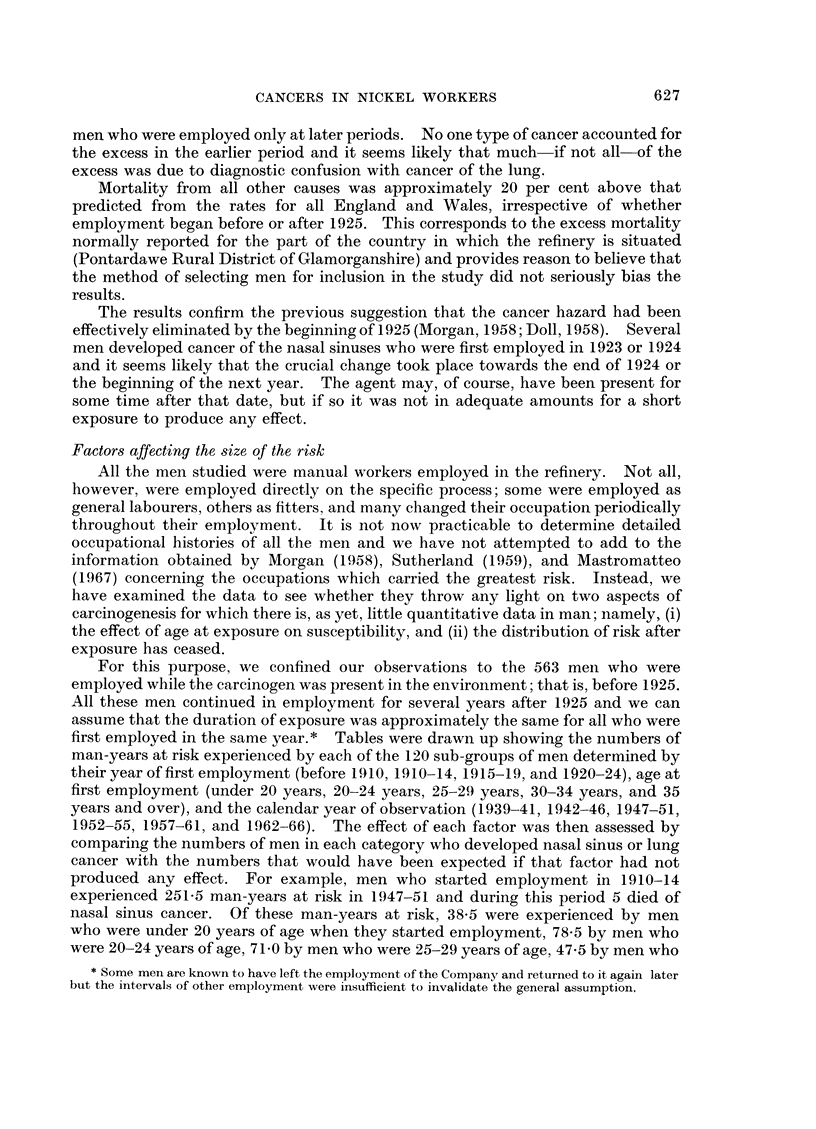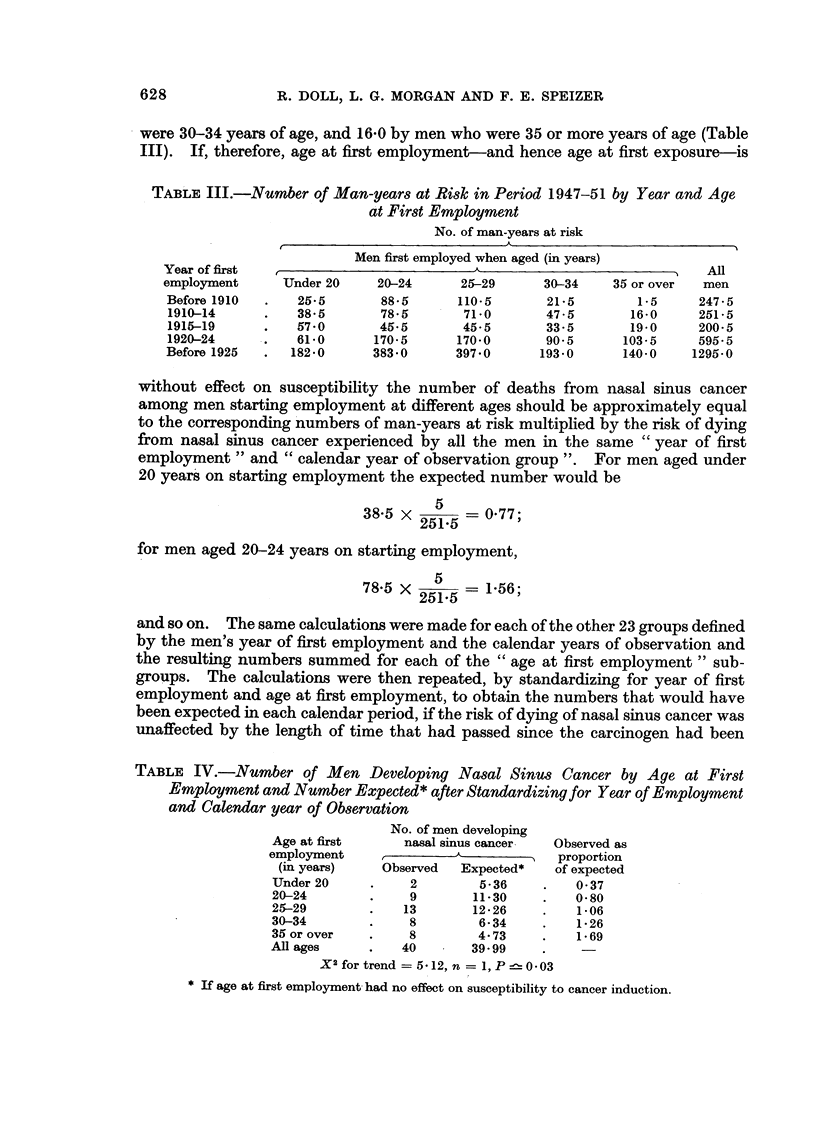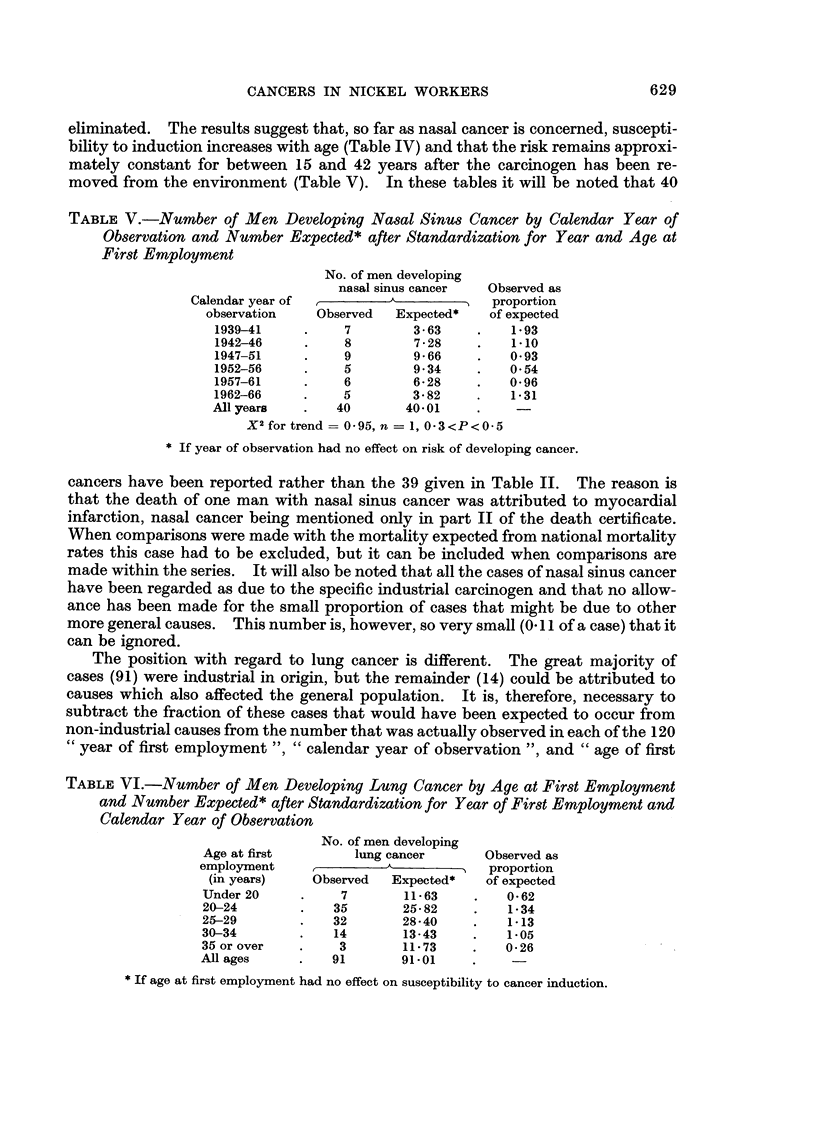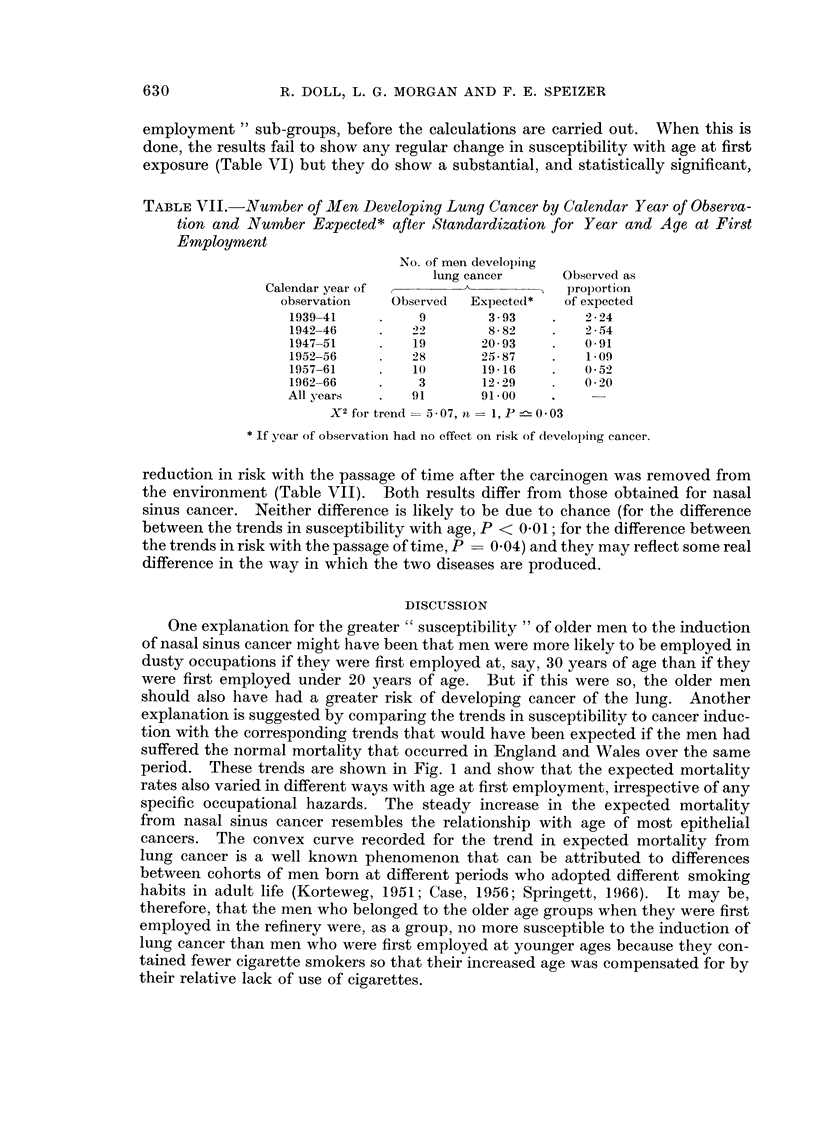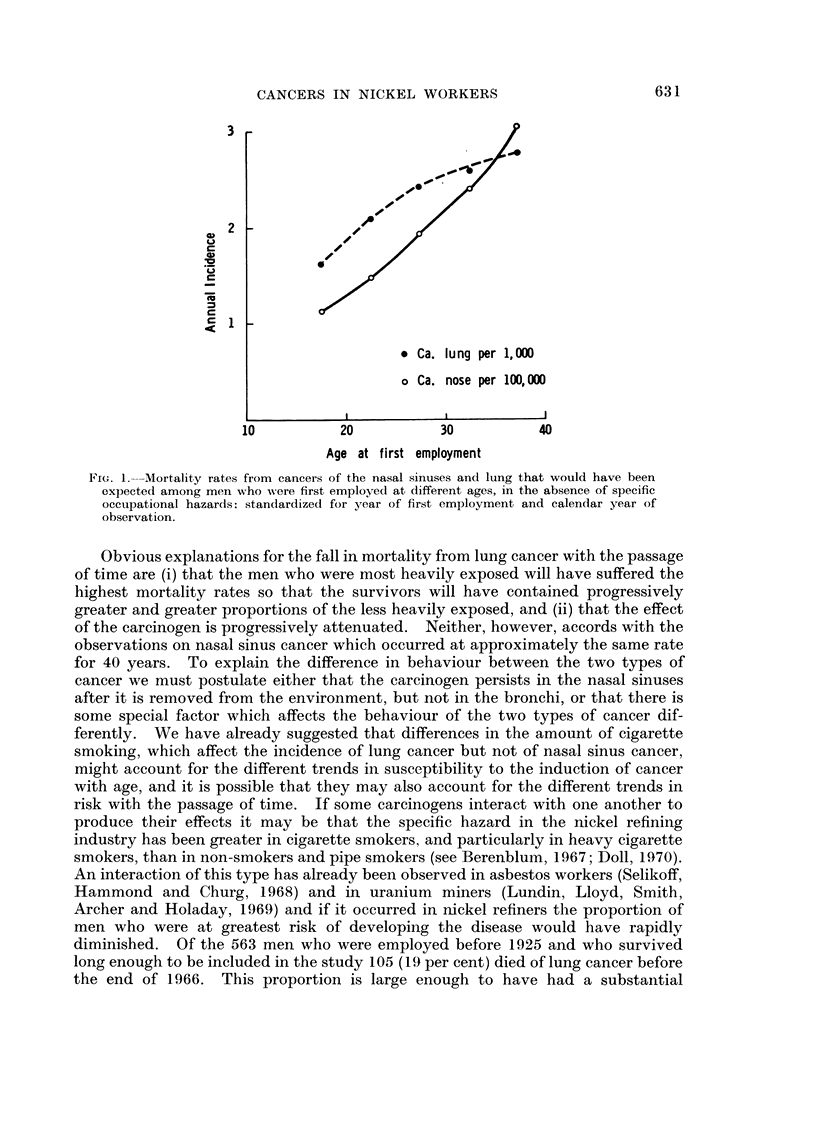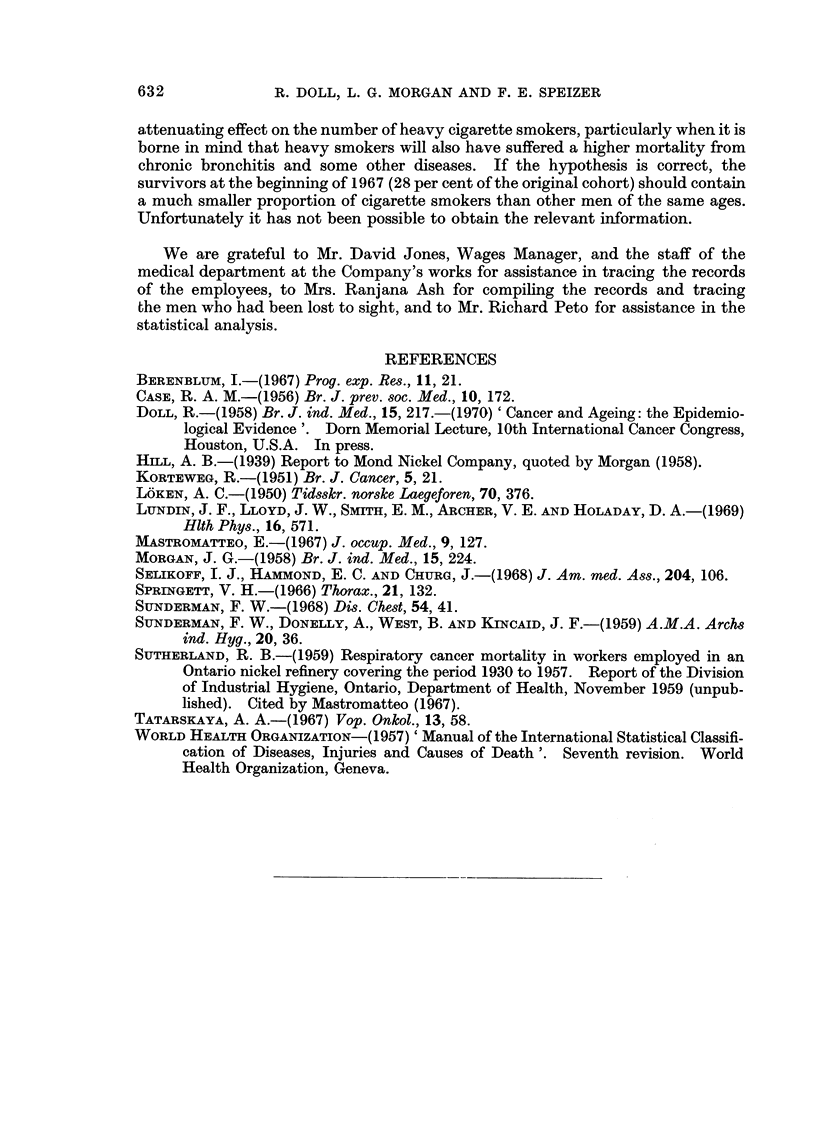# Cancers of the Lung and Nasal Sinuses in Nickel Workers

**DOI:** 10.1038/bjc.1970.76

**Published:** 1970-12

**Authors:** R. Doll, L. G. Morgan, F. E. Speizer

## Abstract

Men employed in a nickel refinery in South Wales were investigated to determine whether the specific risks of developing carcinoma of the bronchi and nasal sinuses, which had been associated with the refining of nickel, are still present. The data obtained were also used to compare the effect of age at exposure on susceptibility to cancer induction and to determine the rate of change of mortality after exposure to a carcinogenic agent had ceased.

Eight hundred and forty five men were studied who had been employed in the industry for at least 5 years and whose first employment was in or before April 1994. All but 27 (3.2 per cent) were traced until death or January 1, 1967.

Altogether 482 of the men had died: 113 from lung cancer and 39 from nasal cancer. In men employed before 1925, deaths from lung cancer varied from about 5 to 10 times the numbers that would have been expected from the corresponding national mortality rates, while the deaths from nasal cancer varied from about 100 to 900 times the expected numbers. Among men first employed in 1925 or after there were 8 deaths from lung cancer against 6.2 expected and no deaths from nasal cancer. The death rate from causes other than cancer was similar to that experienced by men in the same geographical area irrespective of their date of first employment.

Susceptibility to the induction of nasal cancer increased with age at first exposure, but susceptibility to the induction of lung cancer varied irregularly. The trends in susceptibility showed some similarity to the trends in the national mortality among men employed at similar ages. It is suggested that susceptibility to cancer induction is determined by the amount of previous exposure to other agents.

The risk of developing nasal cancer persisted with little change 15 to 42 years after the carcinogen was eliminated whereas the risk of developing lung cancer decreased. If the effects of cigarette smoking and the specific occupational hazard interact, the reduction in the risk of lung cancer could be due to the differential elimination of heavy cigarette smokers.


// BRITISH JOURNAL OF CANCER

VOL. XXIV DECEMBER, 1970 NO. 4

### CANCERS OF THE LUNG AND NASAL SINUSES IN NICKEL WORKERS

R. DOLL, L. G. MORGAN AND F. E. SPEIZER*

*From the Department of the Regius Professor of Medicine, University of Oxford, and the Mond Nickel Company Ltd, Swansea*

Received for publication August 26, 1970

**SUMMARY.—Men employed in a nickel refinery in South Wales were investigated to determine whether the specific risks of developing carcinoma of the bronchi and nasal sinuses, which had been associated with the refining of nickel, are still present. The data obtained were also used to compare the effect of age at exposure on susceptibility to cancer induction and to determine the rate of change of mortality after exposure to a carcinogenic agent had ceased.**

**Eight hundred and forty five men were studied who had been employed in the industry for at least 5 years and whose first employment was in or before April 1944. All but 27 (3·2 per cent) were traced until death or January 1, 1967.**

**Altogether 482 of the men had died: 113 from lung cancer and 39 from nasal cancer. In men employed before 1925, deaths from lung cancer varied from about 5 to 10 times the numbers that would have been expected from the corresponding national mortality rates, while the deaths from nasal cancer varied from about 100 to 900 times the expected numbers. Among men first employed in 1925 or after there were 8 deaths from lung cancer against 6·2 expected and no deaths from nasal cancer. The death rate from causes other than cancer was similar to that experienced by men in the same geographical area irrespective of their date of first employment.**

**Susceptibility to the induction of nasal cancer increased with age at first exposure, but susceptibility to the induction of lung cancer varied irregularly. The trends in susceptibility showed some similarity to the trends in the national mortality among men employed at similar ages. It is suggested that susceptibility to cancer induction is determined by the amount of previous exposure to other agents.**

**The risk of developing nasal cancer persisted with little change 15 to 42 years after the carcinogen was eliminated whereas the risk of developing lung cancer decreased. If the effects of cigarette smoking and the specific occupational hazard interact, the reduction in the risk of lung cancer could be due to the differential elimination of heavy cigarette smokers.**

THE existence of a specific hazard of developing cancer of the nasal sinuses in the nickel industry was suspected in 1927, but was not established until Bradford

* Edward Livingstone Trudeau, Fellow of the American Thoracic Society and Assistant Professor of Medicine, Harvard Medical School, Boston, U.S.A.

Hill reported the results of his investigation to the Mond Nickel Company in 1939. By that time it was clear that the hazard also included the development of cancer of the lung. Ten years later these two types of cancer were prescribed as occupational diseases in Great Britain, when they occurred in men working " in a factory where nickel is produced by decomposition of a gaseous nickel compound "; that is, among men who were employed in a refinery in South Wales, where the final process consisted in the production and subsequent decomposition of nickel carbonyl. By the end of 1957, 131 cases of lung cancer and 62 cases of nasal sinus cancer were known to have occurred in nickel refiners in Wales and detailed evidence concerning the development of these two industrial diseases was published by Morgan (1958) and by Doll (1958). The epidemiological evidence in their reports strongly suggested that the risk was associated with the preliminary steps of the process preceding the formation of nickel carbonyl and that the risk had been eliminated from the industry in Britain by 1925.

Before 1925, a partly refined ore was imported from Canada, which contained sulphides of nickel and copper and a variety of other elements including cobalt, selenium, tellurium, sulphur, and several of the precious metals. Preliminary treatment removed the copper by leaching with an impure form of sulphuric acid containing significant amounts of arsenic. The partially purified material was then subjected to an exceedingly dusty process of calcination. Between 1920 and 1925 considerable changes took place in the refinery; less dusty calciners and some personal protection against dust were introduced and arsenic-free sulphuric acid began to be used. But the essential chemical nature of the process, based on the unique properties of nickel carbonyl, was not altered.

Löken's (1950) observation of 3 cases of lung cancer among a small group of furnace workers in a Norwegian nickel refinery and Tatarskaya's (1967) report of 3 cases of nasal sinus cancer in nickel refiners in the U.S.S.R. supported the conclusion that the hazard was not specifically due to the carbonyl process and the conclusion was confirmed by the demonstration of a substantial hazard of both nasal sinus and bronchial cancer in men employed in dusty occupations such as cupola and furnace work in a refinery in Ontario (Sutherland, 1959; Mastromatteo, 1967). In all these refineries the electrolytic process, and not the carbonyl process, was used.

Sunderman, however, showed that nickel carbonyl was capable of producing pulmonary squamous and adenocarcinomas in rats (Sunderman, Donelly, West and Kincaid, 1959; Sunderman, 1968) and we have, therefore, sought further evidence of the elimination of the risk from the Welsh refinery by examining the mortality experience of cohorts of men employed at different times between 1902 and 1944. We have subsequently used these data to study the effect of age at exposure on susceptibility to cancer induction and the rate of change of mortality after exposure to the agent had ceased.

### METHOD

The men to be studied were identified from data provided by the Company. For many years the Company had used weekly paysheets, on which all men receiving an hourly wage were listed by name and works' reference number, irrespective of whether they had been at work that week or not. Paysheets were inspected for the first week in April of the years 1934, 1939, 1944, and 1949 and all men were included whose names and numbers were recorded on any two of the sheets, unless they were noted on one of the two sheets as having been in the Armed Forces or transferred elsewhere for war work. By this means the population was limited to men who were likely to have been employed for at least 5 years and follow up was facilitated.

The names of all the men included in the study were identified in the Company's register of new employees. This gave the year when they were first employed and much other information that helped them to be traced. From 1902 to 1933 the register of new employees also gave the men's ages; in later years this was sometimes omitted, in which case it had to be obtained from other sources, such as pension records or death certificates.

All the men included in the study were traced back to their year of first employment and all but 27 (3·2 per cent) were followed successfully to the date of their death or the beginning of 1967, whichever was the earlier. The 27 men who were incompletely followed were excluded from the study from the date when they were last known to have been employed. Copies of the death certificates were obtained for all who had died, and the cause of death was classified according to the rules of the International Classification of Causes of Death (World Health Organization, 1957)*.

Because of the method of selection, no man came under observation until 1939. The man-years at risk were, therefore, calculated only for the period 1939 to 1966. The numbers of deaths that would have been expected if the men had suffered the normal mortality experienced in England and Wales as a whole were calculated by multiplying the man-years at risk in each calendar period by the corresponding annual age-specific mortality rates recorded in England and Wales. For nasal cancer age-specific rates were not available before 1950 and the rates for 1950–54 were used for the earlier years. This, however, is unlikely to have introduced any appreciable error, as the crude mortality rate for nasal cancer is known to have remained about the same since the early 1940's.

### RESULTS

### *Disappearance of risk*

Eight hundred and forty-five men fulfilled the selection criteria. The numbers studied and the corresponding man years at risk are shown in Table I, grouped

TABLE I.—*Number of Men and Number of Person-years at Risk, by Year of First Employment in Nickel Industry*

| Group | Year of first employment | No. of men | No. of person-years at risk 1939–66 |
|---|---|---|---|
| 1 | Before 1910 | 96 | 955·5 |
| 2 | 1910–14 | 130 | 1060·5 |
| 3 | 1915–19 | 87 | 915·0 |
| 4 | 1920–24 | 250 | 2923·0 |
| 1 to 4 | Before 1925 | 563 | 5854·0 |
| 5 | 1925–29 | 77 | 1136·0 |
| 6 | 1930–44 | 205 | 2945·0 |
| 5 and 6 | 1925–44 | 282 | 4081·0 |
| All groups | Before 1945 | 845 | 9935·0 |

* The use of these rules for the classification of deaths that occurred before 1959, rather than the ones that were current at the time, has no effect on the numbers of deaths attributed to cancers of the lung and nose.

according to the man's year of entry into the industry. Men in group I started employment from 1902 to 1909, men in groups 2 to 5 started in the succeeding quinquennia, and men in group 6 started in 1930 or later.

Four hundred and eighty-two of the men died before January 1, 1967. The distribution of deaths by cause and year of first employment is shown in Table II.

TABLE II.—*Number of Deaths Observed and Expected* by Year of First Employment in Nickel Industry and Cause of Death*

| Cause of death | Year of first employment | No. of deaths: Observed | No. of deaths: Expected | Observed deaths as proportion of expected |
|---|---|---|---|---|
| Nasal sinus cancer | Before 1910 | 8 | 0·026 | 308 |
| | 1910–14 | 20 | 0·023 | 870 |
| | 1915–19 | 6 | 0·015 | 400 |
| | 1920–24 | 5 | 0·043 | 116 |
| | Before 1925 | 39 | 0·107 | 364 |
| | 1925–29 | 0 | 0·014 | — |
| | 1930–44 | 0 | 0·022 | |
| Lung cancer | Before 1910 | 20 | 2·11 | 9·5 |
| | 1910–14 | 29 | 2·75 | 10·5 |
| | 1915–19 | 13 | 2·29 | 5·7 |
| | 1920–24 | 43 | 6·79 | 6·3 |
| | Before 1925 | 105 | 13·94 | 7·5 |
| | 1925–29 | 4 | 2·27 | 1·3 |
| | 1930–44 | 4 | 3·79 | |
| Other neoplasms | Before 1910 | 9 | 8·43 | 1·1 |
| | 1910–14 | 10 | 7·18 | 1·4 |
| | 1915–19 | 9 | 4·32 | 2·1 |
| | 1920–24 | 21 | 11·57 | 1·8 |
| | Before 1925 | 49 | 31·50 | 1·6 |
| | 1925–29 | 3 | 3·67 | 1·0 |
| | 1930–44 | 6 | 5·49 | |
| All other causes | Before 1910 | 49 | 50·86 | 1·0 |
| | 1910–14 | 48 | 40·98 | 1·2 |
| | 1915–19 | 31 | 23·33 | 1·3 |
| | 1920–24 | 86 | 62·01 | 1·4 |
| | Before 1925 | 214 | 177·18 | 1·2 |
| | 1925–29 | 20 | 20·06 | 1·2 |
| | 1930–44 | 38 | 28·43 | |
| All causes | Before 1910 | 86 | 61·43 | 1·4 |
| | 1910–14 | 107 | 50·94 | 2·1 |
| | 1915–19 | 59 | 29·94 | 2·0 |
| | 1920–24 | 155 | 80·41 | 1·9 |
| | Before 1925 | 407 | 222·72 | 1·8 |
| | 1925–29 | 27 | 26·01 | 1·2 |
| | 1930–44 | 48 | 37·73 | |

* If mortality had been the same as in all men of the same ages in England and Wales over the same period.

Men who started employment before 1925 suffered a mortality from nasal cancer that varied from about 100 to 900 times the national average. No deaths from this cancer occurred in men who started in 1925 or later. The mortality from lung cancer followed the same pattern, the observed mortality being 5 to 10 times that expected in men who started employment before 1925 and close to that expected for men who started later.

The mortality from other cancers was also slightly increased among men employed before 1925 (49 deaths against 31·50 expected, $P < 0·01$) but not among men who were employed only at later periods. No one type of cancer accounted for the excess in the earlier period and it seems likely that much—if not all—of the excess was due to diagnostic confusion with cancer of the lung.

Mortality from all other causes was approximately 20 per cent above that predicted from the rates for all England and Wales, irrespective of whether employment began before or after 1925. This corresponds to the excess mortality normally reported for the part of the country in which the refinery is situated (Pontardawe Rural District of Glamorganshire) and provides reason to believe that the method of selecting men for inclusion in the study did not seriously bias the results.

The results confirm the previous suggestion that the cancer hazard had been effectively eliminated by the beginning of 1925 (Morgan, 1958; Doll, 1958). Several men developed cancer of the nasal sinuses who were first employed in 1923 or 1924 and it seems likely that the crucial change took place towards the end of 1924 or the beginning of the next year. The agent may, of course, have been present for some time after that date, but if so it was not in adequate amounts for a short exposure to produce any effect.

*Factors affecting the size of the risk*

All the men studied were manual workers employed in the refinery. Not all, however, were employed directly on the specific process; some were employed as general labourers, others as fitters, and many changed their occupation periodically throughout their employment. It is not now practicable to determine detailed occupational histories of all the men and we have not attempted to add to the information obtained by Morgan (1958), Sutherland (1959), and Mastromatteo (1967) concerning the occupations which carried the greatest risk. Instead, we have examined the data to see whether they throw any light on two aspects of carcinogenesis for which there is, as yet, little quantitative data in man; namely, (i) the effect of age at exposure on susceptibility, and (ii) the distribution of risk after exposure has ceased.

For this purpose, we confined our observations to the 563 men who were employed while the carcinogen was present in the environment; that is, before 1925. All these men continued in employment for several years after 1925 and we can assume that the duration of exposure was approximately the same for all who were first employed in the same year.* Tables were drawn up showing the numbers of man-years at risk experienced by each of the 120 sub-groups of men determined by their year of first employment (before 1910, 1910–14, 1915–19, and 1920–24), age at first employment (under 20 years, 20–24 years, 25–29 years, 30–34 years, and 35 years and over), and the calendar year of observation (1939–41, 1942–46, 1947–51, 1952–55, 1957–61, and 1962–66). The effect of each factor was then assessed by comparing the numbers of men in each category who developed nasal sinus or lung cancer with the numbers that would have been expected if that factor had not produced any effect. For example, men who started employment in 1910–14 experienced 251·5 man-years at risk in 1947–51 and during this period 5 died of nasal sinus cancer. Of these man-years at risk, 38·5 were experienced by men who were under 20 years of age when they started employment, 78·5 by men who were 20–24 years of age, 71·0 by men who were 25–29 years of age, 47·5 by men who

* Some men are known to have left the employment of the Company and returned to it again later but the intervals of other employment were insufficient to invalidate the general assumption.

were 30–34 years of age, and 16·0 by men who were 35 or more years of age (Table III). If, therefore, age at first employment—and hence age at first exposure—is

TABLE III.—*Number of Man-years at Risk in Period* 1947–51 *by Year and Age at First Employment*

| Year of first employment | No. of man-years at risk | | | | | |
|---|---|---|---|---|---|---|
| | Men first employed when aged (in years) | | | | | All men |
| | Under 20 | 20–24 | 25–29 | 30–34 | 35 or over | |
| Before 1910 . | 25·5 | 88·5 | 110·5 | 21·5 | 1·5 | 247·5 |
| 1910–14 . | 38·5 | 78·5 | 71·0 | 47·5 | 16·0 | 251·5 |
| 1915–19 . | 57·0 | 45·5 | 45·5 | 33·5 | 19·0 | 200·5 |
| 1920–24 . | 61·0 | 170·5 | 170·0 | 90·5 | 103·5 | 595·5 |
| Before 1925 . | 182·0 | 383·0 | 397·0 | 193·0 | 140·0 | 1295·0 |

without effect on susceptibility the number of deaths from nasal sinus cancer among men starting employment at different ages should be approximately equal to the corresponding numbers of man-years at risk multiplied by the risk of dying from nasal sinus cancer experienced by all the men in the same " year of first employment " and " calendar year of observation group ". For men aged under 20 years on starting employment the expected number would be

$$38{\cdot}5 \times \frac{5}{251{\cdot}5} = 0{\cdot}77;$$

for men aged 20–24 years on starting employment,

$$78{\cdot}5 \times \frac{5}{251{\cdot}5} = 1{\cdot}56;$$

and so on. The same calculations were made for each of the other 23 groups defined by the men's year of first employment and the calendar years of observation and the resulting numbers summed for each of the " age at first employment " subgroups. The calculations were then repeated, by standardizing for year of first employment and age at first employment, to obtain the numbers that would have been expected in each calendar period, if the risk of dying of nasal sinus cancer was unaffected by the length of time that had passed since the carcinogen had been

TABLE IV.—*Number of Men Developing Nasal Sinus Cancer by Age at First Employment and Number Expected* after Standardizing for Year of Employment and Calendar year of Observation*

| Age at first employment (in years) | No. of men developing nasal sinus cancer | | Observed as proportion of expected |
|---|---|---|---|
| | Observed | Expected* | |
| Under 20 . | 2 | 5·36 . | 0·37 |
| 20–24 . | 9 | 11·30 . | 0·80 |
| 25–29 . | 13 | 12·26 . | 1·06 |
| 30–34 . | 8 | 6·34 . | 1·26 |
| 35 or over . | 8 | 4·73 . | 1·69 |
| All ages . | 40 | 39·99 . | — |

$X^2$ for trend $= 5{\cdot}12$, $n = 1$, $P \simeq 0{\cdot}03$

* If age at first employment had no effect on susceptibility to cancer induction.

eliminated. The results suggest that, so far as nasal cancer is concerned, susceptibility to induction increases with age (Table IV) and that the risk remains approximately constant for between 15 and 42 years after the carcinogen has been removed from the environment (Table V). In these tables it will be noted that 40

TABLE V.—*Number of Men Developing Nasal Sinus Cancer by Calendar Year of Observation and Number Expected* after Standardization for Year and Age at First Employment*

| Calendar year of observation | No. of men developing nasal sinus cancer: Observed | Expected* | Observed as proportion of expected |
|---|---|---|---|
| 1939–41 | 7 | 3·63 | 1·93 |
| 1942–46 | 8 | 7·28 | 1·10 |
| 1947–51 | 9 | 9·66 | 0·93 |
| 1952–56 | 5 | 9·34 | 0·54 |
| 1957–61 | 6 | 6·28 | 0·96 |
| 1962–66 | 5 | 3·82 | 1·31 |
| All years | 40 | 40·01 | — |

$X^2$ for trend $= 0\cdot95$, $n = 1$, $0\cdot3 < P < 0\cdot5$

* If year of observation had no effect on risk of developing cancer.

cancers have been reported rather than the 39 given in Table II. The reason is that the death of one man with nasal sinus cancer was attributed to myocardial infarction, nasal cancer being mentioned only in part II of the death certificate. When comparisons were made with the mortality expected from national mortality rates this case had to be excluded, but it can be included when comparisons are made within the series. It will also be noted that all the cases of nasal sinus cancer have been regarded as due to the specific industrial carcinogen and that no allowance has been made for the small proportion of cases that might be due to other more general causes. This number is, however, so very small (0·11 of a case) that it can be ignored.

The position with regard to lung cancer is different. The great majority of cases (91) were industrial in origin, but the remainder (14) could be attributed to causes which also affected the general population. It is, therefore, necessary to subtract the fraction of these cases that would have been expected to occur from non-industrial causes from the number that was actually observed in each of the 120 " year of first employment ", " calendar year of observation ", and " age of first

TABLE VI.—*Number of Men Developing Lung Cancer by Age at First Employment and Number Expected* after Standardization for Year of First Employment and Calendar Year of Observation*

| Age at first employment (in years) | No. of men developing lung cancer: Observed | Expected* | Observed as proportion of expected |
|---|---|---|---|
| Under 20 | 7 | 11·63 | 0·62 |
| 20–24 | 35 | 25·82 | 1·34 |
| 25–29 | 32 | 28·40 | 1·13 |
| 30–34 | 14 | 13·43 | 1·05 |
| 35 or over | 3 | 11·73 | 0·26 |
| All ages | 91 | 91·01 | — |

* If age at first employment had no effect on susceptibility to cancer induction.

employment " sub-groups, before the calculations are carried out. When this is done, the results fail to show any regular change in susceptibility with age at first exposure (Table VI) but they do show a substantial, and statistically significant,

TABLE VII.—*Number of Men Developing Lung Cancer by Calendar Year of Observation and Number Expected* after Standardization for Year and Age at First Employment*

| Calendar year of observation | No. of men developing lung cancer: Observed | Expected* | Observed as proportion of expected |
|---|---|---|---|
| 1939–41 | 9 | 3·93 | 2·24 |
| 1942–46 | 22 | 8·82 | 2·54 |
| 1947–51 | 19 | 20·93 | 0·91 |
| 1952–56 | 28 | 25·87 | 1·09 |
| 1957–61 | 10 | 19·16 | 0·52 |
| 1962–66 | 3 | 12·29 | 0·20 |
| All years | 91 | 91·00 | — |

$X^2$ for trend $= 5 \cdot 07$, $n = 1$, $P \simeq 0 \cdot 03$

* If year of observation had no effect on risk of developing cancer.

reduction in risk with the passage of time after the carcinogen was removed from the environment (Table VII). Both results differ from those obtained for nasal sinus cancer. Neither difference is likely to be due to chance (for the difference between the trends in susceptibility with age, $P < 0{\cdot}01$; for the difference between the trends in risk with the passage of time, $P = 0{\cdot}04$) and they may reflect some real difference in the way in which the two diseases are produced.

### DISCUSSION

One explanation for the greater " susceptibility " of older men to the induction of nasal sinus cancer might have been that men were more likely to be employed in dusty occupations if they were first employed at, say, 30 years of age than if they were first employed under 20 years of age. But if this were so, the older men should also have had a greater risk of developing cancer of the lung. Another explanation is suggested by comparing the trends in susceptibility to cancer induction with the corresponding trends that would have been expected if the men had suffered the normal mortality that occurred in England and Wales over the same period. These trends are shown in Fig. 1 and show that the expected mortality rates also varied in different ways with age at first employment, irrespective of any specific occupational hazards. The steady increase in the expected mortality from nasal sinus cancer resembles the relationship with age of most epithelial cancers. The convex curve recorded for the trend in expected mortality from lung cancer is a well known phenomenon that can be attributed to differences between cohorts of men born at different periods who adopted different smoking habits in adult life (Korteweg, 1951; Case, 1956; Springett, 1966). It may be, therefore, that the men who belonged to the older age groups when they were first employed in the refinery were, as a group, no more susceptible to the induction of lung cancer than men who were first employed at younger ages because they contained fewer cigarette smokers so that their increased age was compensated for by their relative lack of use of cigarettes.

FIG. 1.—Mortality rates from cancers of the nasal sinuses and lung that would have been expected among men who were first employed at different ages, in the absence of specific occupational hazards: standardized for year of first employment and calendar year of observation.

Obvious explanations for the fall in mortality from lung cancer with the passage of time are (i) that the men who were most heavily exposed will have suffered the highest mortality rates so that the survivors will have contained progressively greater and greater proportions of the less heavily exposed, and (ii) that the effect of the carcinogen is progressively attenuated. Neither, however, accords with the observations on nasal sinus cancer which occurred at approximately the same rate for 40 years. To explain the difference in behaviour between the two types of cancer we must postulate either that the carcinogen persists in the nasal sinuses after it is removed from the environment, but not in the bronchi, or that there is some special factor which affects the behaviour of the two types of cancer differently. We have already suggested that differences in the amount of cigarette smoking, which affect the incidence of lung cancer but not of nasal sinus cancer, might account for the different trends in susceptibility to the induction of cancer with age, and it is possible that they may also account for the different trends in risk with the passage of time. If some carcinogens interact with one another to produce their effects it may be that the specific hazard in the nickel refining industry has been greater in cigarette smokers, and particularly in heavy cigarette smokers, than in non-smokers and pipe smokers (see Berenblum, 1967; Doll, 1970). An interaction of this type has already been observed in asbestos workers (Selikoff, Hammond and Churg, 1968) and in uranium miners (Lundin, Lloyd, Smith, Archer and Holaday, 1969) and if it occurred in nickel refiners the proportion of men who were at greatest risk of developing the disease would have rapidly diminished. Of the 563 men who were employed before 1925 and who survived long enough to be included in the study 105 (19 per cent) died of lung cancer before the end of 1966. This proportion is large enough to have had a substantial attenuating effect on the number of heavy cigarette smokers, particularly when it is borne in mind that heavy smokers will also have suffered a higher mortality from chronic bronchitis and some other diseases. If the hypothesis is correct, the survivors at the beginning of 1967 (28 per cent of the original cohort) should contain a much smaller proportion of cigarette smokers than other men of the same ages. Unfortunately it has not been possible to obtain the relevant information.

We are grateful to Mr. David Jones, Wages Manager, and the staff of the medical department at the Company's works for assistance in tracing the records of the employees, to Mrs. Ranjana Ash for compiling the records and tracing the men who had been lost to sight, and to Mr. Richard Peto for assistance in the statistical analysis.

### REFERENCES

BERENBLUM, I.—(1967) *Prog. exp. Res.*, **11**, 21.
CASE, R. A. M.—(1956) *Br. J. prev. soc. Med.*, **10**, 172.
DOLL, R.—(1958) *Br. J. ind. Med.*, **15**, 217.—(1970) 'Cancer and Ageing: the Epidemiological Evidence'. Dorn Memorial Lecture, 10th International Cancer Congress, Houston, U.S.A. In press.
HILL, A. B.—(1939) Report to Mond Nickel Company, quoted by Morgan (1958).
KORTEWEG, R.—(1951) *Br. J. Cancer*, **5**, 21.
LÖKEN, A. C.—(1950) *Tidsskr. norske Laegeforen*, **70**, 376.
LUNDIN, J. F., LLOYD, J. W., SMITH, E. M., ARCHER, V. E. AND HOLADAY, D. A.—(1969) *Hlth Phys.*, **16**, 571.
MASTROMATTEO, E.—(1967) *J. occup. Med.*, **9**, 127.
MORGAN, J. G.—(1958) *Br. J. ind. Med.*, **15**, 224.
SELIKOFF, I. J., HAMMOND, E. C. AND CHURG, J.—(1968) *J. Am. med. Ass.*, **204**, 106.
SPRINGETT, V. H.—(1966) *Thorax.*, **21**, 132.
SUNDERMAN, F. W.—(1968) *Dis. Chest*, **54**, 41.
SUNDERMAN, F. W., DONELLY, A., WEST, B. AND KINCAID, J. F.—(1959) *A.M.A. Archs ind. Hyg.*, **20**, 36.
SUTHERLAND, R. B.—(1959) Respiratory cancer mortality in workers employed in an Ontario nickel refinery covering the period 1930 to 1957. Report of the Division of Industrial Hygiene, Ontario, Department of Health, November 1959 (unpublished). Cited by Mastromatteo (1967).
TATARSKAYA, A. A.—(1967) *Vop. Onkol.*, **13**, 58.
WORLD HEALTH ORGANIZATION—(1957) 'Manual of the International Statistical Classification of Diseases, Injuries and Causes of Death'. Seventh revision. World Health Organization, Geneva.